# Hema-Uro-Chrome—A New Laboratory Test for Cancer and Sarcoma; from the Urine

**Published:** 1914-01

**Authors:** 


HEMA-URO-CHROME—A NEW LABORATORY TEST FOR CANCER AND SARCOMA; FROM THE URINE. Theodore G. Davis, Ph. G., M. D., Los Angeles. *California State Journal of Medicine*, Oct., 1913. The urine should be carefully collected, fresh, no preservative should be added; unless when it is impossible to make immediate examination, Hydrochloric acid in the proportion of 1 part to 10 of urine may be added, this being the proportion used in the test.

Formaldehyde inhibits the test; and hexamethylin tetramin, formin or urotropin should be avoided where the test is to be applied.

After many experiments extending over considerable time, I determined the following to be the most satisfactory method of procedure. (While the quantity may vary, the proportions should be maintained.) Select a flat bottomed flask of about 180 cc.—or 6 fl. ozs. capacity, with a narrow neck that the ether may be brought up into it, easily seen and separated.

To 100 cc. of urine in the flask add 10 cc. of hydrochloric acid.

Heat over a slow fire until ebullition begins; turn out the fire, and allow it to cool slowly for a time, after which cooling may be hastened by immersion in water. When cold add 30 cc. of ether, cork, tieing the cork to prevent evaporation. Turn the flask upside down several times during the six or eight hours required to complete the test. Avoid hard shaking which interferes with separation of the ether. While in cases of pronounced or extensive cancer the ether will acquire a markedly red color in as short a time as twenty minutes, I have found six or eight hours required for the complete extraction of the hema-uro-chrome by the ether. By the addition of cold water, the colored ether may be raised into the neck of the flask for observation, and be removed by a pipet into a bottle, corked, sealed and kept for comparison, if desired.

As a certain amount of the ether remains in solution by the contents of the flask, and some is lost by evaporation, I have depended upon the relative depth of color extracted by a certain quantity of ether from a definite amount of urine and hydrochloric acid, by the process described.

It might be possible by adding ether to the contents of the flask to replace that lost, making the quantity removed up to 30 cc. and make colorimetric comparisons, as is done in other color tests; but I doubt if it would be of any great value, for when some of the ethereal solution is allowed to evaporate in a white dish, the red hema-uro-chrome will be seen upon the upper portion, indican, when present, as an idigo-blue ring, slightly lower, while bile-acids and coloring remain in the bottom as a sticky brownish yellow mass. These in varying proportions must of necessity produce different tints; beside which the cleavage of hemoglobin into globulin and the several cleavage products of hematin, of which there may be at least three containing iron, and several not containing iron; indican, bile-acids and coloring, at times fatty substances of the amino-acid group, as well as crystals of ammonium chloride and urate, all of which modify the color of the ethereal solution. While the latter have pathologic significance, this simple test yields us three substances of considerable significance when in excess, viz., indican, bile-acids and coloring, and the hema-urochrome. That these represent a pathological physiology, there can be no doubt. The red hema-uro-chrome of cancer is so pronounced, it astonishes the beginner; and occurs even with small growths not otherwise discernable.

Herein lies the great value of this test which enables us to make an early diagnosis, and apply treatment at a time when there is hope of cure.

That this hema-uro-chrome is produced by cleavage of hemaglobin by a product of the cancer cell, probably an enzyme, there is little doubt; but as the chemistry of the uro-chromes is so complicated, and statements concerning them so conflicting, I leave this for future investigation.

I am not aware of the previous application of the uro-chromes in the diagnosis of disease; except Erlich's aldehyde test for insufficiency of the hepatic cells, which should be applied to the urine under examination for cancer. (It is prepared and used as follows: Para-dimethylaminobenzylaldehyde 4; alcohol 16; water to 200. A few drops in 4 cc. of urine, when heated, becomes cherry-red if hepatic insufficiency exists.) This color is quite different from the red hema-uro-chrome of cancer. It aids in the elimination of cirrhosis of the liver, which is one of the confusing disease factors in all sero-diagnostic tests for cancer. Beside this I have found syncytioma, which is practically a malignant condition, and extensive suppurating processes, especially if tubercular, to give a somewhat red color to the ether, but not to compare with that given by cancer. A pink tint of more or less depth will occur when blood is in the urine from any cause; also in the urine of persons having malaria, "tick-fever" or Babesia, the several infections due to spirillum; "hookworm" and other intestinal parasites, as well as the primary and secondary anemias; but none of these give color comparable with that from the urine of a cancer patient, and should be readily eliminated.

It is well to remember that all laboratory tests are suggestive or confirmatory and liable to a percentage of errors, yet this test has proven positive in a larger percentage of cases than any other with which I am acquainted; even when the cancer was very small and unsuspected, not determinable by palpation or other diagnostic method. Again a negative finding will enable us to relieve that mental distress associated with a suspicion of cancer or of its recurrence.

